# Interprofessional diagnostic management teams: a scoping review protocol

**DOI:** 10.1186/s13643-023-02391-2

**Published:** 2023-11-22

**Authors:** Nicoline Lykke Hansen, Helle Precht, Palle Larsen, Lene Noehr-Jensen

**Affiliations:** 1 Biomedical Laboratory Science, UCL University College, Niels Bohrs Allé 1, 5230 Odense S, Denmark; 2 Health Sciences Research Centre, UCL University College, Niels Bohrs Allé 1, 5230 Odense S, Denmark; 3 Department of Regional Health Research, University of Southern Denmark, Odense, Denmark; 4 Department of Radiology, Kolding, Lillebaelt Hospital, University Hospitals of Southern Denmark, Odense, Denmark

**Keywords:** Diagnostic error, Diagnostic excellence, Diagnostic safety, Interprofessional teamwork, Patient safety

## Abstract

**Background:**

Diagnostic errors are a major problem in healthcare. In 2015, the report “Improving Diagnosis in Health Care” by the National Academies of Sciences, Engineering, and Medicine (NASEM) stated that it is likely that most people will experience at least one diagnostic error in their lifetime. The report suggests implementing diagnostic management teams, including patients and their relatives, diagnosticians, and healthcare professionals who support the diagnostic process, to limit diagnostic error and improve patient safety. Implementing interprofessional diagnostic management teams (IDMT), however, is not an easy task due to the complexity of the diagnostic processes and the traditional organization of healthcare with divided departments and healthcare professional who operate in different geographic locations. As this topic is still emerging, a scoping review is ideal to determine the scope of the body of literature on IDMT, indicate the volume of literature and studies available and identify any gaps in knowledge. In a long-term perspective, this scoping review will contribute to prevent diagnostic errors and improve patient safety, for adults and children with physical health issues.

**Methods:**

We will conduct this scoping review in accordance with the JBI methodology and report it based on the PRISMA-ScR. We will systematically search six databases (EMBASE, PubMed, CINAHL, Academic Search Premier, SCOPUS and Web of Science) for papers published between 1985 and 2023 that describe the use of interprofessional diagnostic management teams. The participants included will be adults and children seeking diagnostic care for physical health issues. The concept studied will be interprofessional diagnostic management teams, and the context will be the diagnostic process in the healthcare system. Studies examining the diagnostic process in psychiatry, odontology or complementary medicine will be excluded. Data extraction, including key study characteristics and findings, will be done by two reviewers independently. Any disagreement will be resolved by discussion and eventually by including the two remainder reviewers.

**Discussion:**

To our knowledge, this will be the first scoping review regarding IDMT and the derived effects on diagnostic safety and can therefore be a very important contribution to improve patient safety significantly during the diagnostic process.

**Protocol registration:**

The project is registered at Open Science Framework (OSF) with ID: osf.io/kv2n6.

**Supplementary Information:**

The online version contains supplementary material available at 10.1186/s13643-023-02391-2.

## Background

Diagnostic errors are a major problem in healthcare. In 2015, the report “Improving Diagnosis in Health Care” by the National Academies of Sciences, Engineering, and Medicine (NASEM) stated that it is likely that most people will experience at least one diagnostic error in their lifetime. Diagnostic errors may cause harm to patients by preventing or delaying appropriate treatment, providing unnecessary or harmful treatment, or resulting in psychological or financial repercussions [1]. The report concluded that improving the diagnostic process is not only possible, but also represents a moral, professional, and public health imperative and address eight goals to achieve this. In recognition that the diagnostic process is a dynamic team-based activity, the first of these goals are “Facilitate more effective teamwork in the diagnostic process amongst health care professionals, patients, and their families”. Health care organizations are recommended to facilitate and support intra- and interprofessional teamwork in the diagnostic process [1]. The report suggests implementing diagnostic management teams, including patients and their relatives, diagnosticians, and healthcare professionals who support the diagnostic process, to limit diagnostic error and improve patient safety [1].

Diagnostic errors are a worldwide challenge. According to the Danish report “Improving Diagnosis in Danish Healthcare” (2019), the diagnostic process has been a blind spot in relation to patient safety in Denmark [2]; an English resume of the report is available from the website of the Danish Society for Patient Safety [3]. An evaluation of cases concerning treatment injuries from 2009 to 2018 shows that 14.5% (13,000 out of 90,000) of all settled cases in Denmark are related to diagnostic errors. Furthermore, the number of cases resulting in death are almost twice as high (1.8 times) in cases related to diagnostic errors, compared to other types of treatment errors. This report also emphasizes the importance of including a broad range of healthcare professionals as well as the patients and relatives in the diagnostic process [2].

Implementing interprofessional diagnostic management teams (IDMT), however, is not an easy task due to the complexity of the diagnostic processes and the traditional organization of healthcare with divided departments and healthcare professionals who operate in different geographic locations. Graber et al*.* describe the challenges in physicians being solely responsible for the diagnostic process in “The new diagnostic team” and explore the roles that different healthcare professionals can play in a dynamic, interprofessional model of the diagnostic team, envisioned in the NASEM report [4].

To advance research and development in this field, a comprehensive view of present knowledge is needed. A preliminary search of MEDLINE, EMBASE and PROSPERO revealed that the available literature regarding IDMT is scarce and still emerging. The preliminary search identified no current or underway systematic reviews or scoping reviews on this topic. As this topic is still emerging, a scoping review is ideal to determine the scope of the body of literature on IDMT, indicate the volume of literature and studies available and identify any gaps in knowledge. The scoping review further aims to identify and map the types of available evidence, clarify key concepts/definitions in the literature and examine how research is conducted in this field [5–7].

This scoping review will shed an important and needed light on the knowledge of IDMT and diagnostic errors. This will provide a solid foundation for further research and development in this significant field. The results will contribute to the crucial work of improving the diagnostic process, by identifying the types of available evidence, clarify key concepts and identify knowledge gaps in IDMT. This will provide an overall picture of the current state of the evidence in the field and identify and highlight knowledge gaps in the area. Furthermore, it will contribute to inform a model for IDMT. In a long-term perspective, this scoping review will contribute to prevent diagnostic errors and improve patient safety, for adults and children with physical health issues.

As there is no formal consensus on notions and definitions in this area, we will for clarity, use the following definitions:

*Diagnostic process* — “ … a complex, patient centered, collaborative activity that involves information gathering and clinical reasoning with the goal of determining a patient’s health problem. This process occurs over time, within the context of a larger healthcare work system that influences the diagnostic process” from “Improving Diagnosis in Health Care” [1].

*Diagnostic error* — “ … the failure to (a) establish an accurate and timely explanation of the patient’s health problem(s) or (b) communicate that explanation to the patient … A diagnosis is not accurate if it differs from the true condition a patient has (or does not have) or if it is imprecise and incomplete (lacking in sufficient detail)… Timeliness means that the diagnosis was not meaningfully delayed” from “Improving Diagnosis in Health Care” [1].

*Multi-disciplinary teams (MDT)* — this term refers to a team of health professionals who have a particular expertise in a specific disease, e.g., breast cancer. MDT is often used in oncology and manages the diagnosis, treatment, and care of oncology patients. Members of the MDTs are mostly doctors from designated specialties, i.e., pathologists, radiologists, surgeons, and oncologists and specially trained nurses [4, 8].

*Interprofessional diagnostic management teams (IDMT)* — a group of diagnostic experts including patients, relatives, doctors, biomedical laboratory scientists, nurses, radiographers, occupational therapists, physiotherapists, medical librarians, or information scientists that team up to support clinical colleagues who are in direct contact with patients. These teams provide their expertise in the diagnostic process. Authors own definition based on Verna et al. (2019) [9], Graber et al. (2017) [4] and Wade et al. (2020) [10].

*Diagnostic excellence* — “ … a systems-level state that effectively integrates health care knowledge, skills, and resources for continuous and measurable improvement of diagnosis, and reduction of risk or occurrence of diagnostic errors, while continuing to meet overall needs of patients and health systems” from “Bringing the clinical laboratory into the strategy to advance diagnostic excellence” from Lubin et al. (2021) [11].

*Mental, behavioural, or neurodevelopmental disorders* — “Mental, behavioural and neurodevelopmental disorders are syndromes characterised by clinically significant disturbance in an individual's cognition, emotional regulation, or behaviour that reflects a dysfunction in the psychological, biological, or developmental processes that underlie mental and behavioural functioning. These disturbances are usually associated with distress or impairment in personal, family, social, educational, occupational, or other important areas of functioning” from World Health Organization: “International Classification of Diseases, Eleventh Revision (ICD-11)” [12].

## Review question

The questions of this scoping review are:

- What is the extent and nature of the present knowledge of IDMT amongst adults and children seeking diagnostic care for a physical health issue in the healthcare system, in a qualitative and quantitative perspective?
- What are the frameworks and models of diagnostic management teams and which patients, relatives and health professionals are involved?
- Has the impact of IDMT been assessed and if so, how?
- Which types of errors or adverse events occur in the diagnostic process?

## Methods

The proposed scoping review will be conducted in accordance with the JBI methodology for scoping reviews [5, 6] and reported based on the Preferred Reporting Items for Systematic Reviews and Meta-analyses extension for scoping review (PRISMA-ScR) [10]. A populated checklist for scoping review protocols recommended by Peters et al*.* [5] are available in Additional file 1.

### Protocol and registration

The project is registered at Open Science Framework (OSF) with ID: osf.io/kv2n6.

### Eligibility criteria

#### Participants

The patients included will be adults and children seeking diagnostic care for a physical health issues.

We will exclude studies examining the diagnostic process of mental, behavioural, or neurodevelopmental disorders as there is a rather sharp division in both organisation and mode of action between the two sections of the health care system in most countries. However, there seems to be an increasing interest in IDMT in the field of mental health disorders, which makes this an obvious subject for another scoping review.

#### Concept

The concept examined by this scoping review consists of IDMT in relation to prevention of diagnostic errors and achieving diagnostic excellence. Studies of all aspects of the diagnostic process will be considered.

This scoping review will consider inclusion of studies that involve patients, relatives, doctors, biomedical laboratory scientists, nurses, radiographers, occupational therapists, physiotherapists, medical librarians, or information scientists in the IDMT. Any combination of these groups is of interest, but an IDMT must consist of at least two professions.

#### Context

This scoping review will include studies of the diagnostic process in the healthcare system.

Studies examining the diagnostic process in psychiatry, odontology, or complementary medicine will be excluded, due to the geographical distances and organizational differences.

#### Types of evidence sources

This scoping review will consider both experimental and quasi-experimental study designs including randomized controlled trials, non-randomized controlled trials, and before and after studies. In addition, analytical observational studies including prospective and retrospective cohort studies, case–control studies, and analytical cross-sectional studies will be considered for inclusion. This review will also consider descriptive observational study designs including case series and descriptive cross-sectional studies for inclusion.

Qualitative studies focusing on qualitative data including, but not limited to, designs such as phenomenology, grounded theory, ethnography, qualitative description, and action research will also be considered for the scoping review.

To identify and map all the types of available and emerging evidence in this field, text, opinion papers, websites, guidelines, policy documents and letters to editors will be identified and considered for inclusion by ultimo 2023/primo 2024, after the systematic literature process.

### Search strategy

The search strategy will aim to locate both published and unpublished studies. A three-step search strategy will be utilized in this review. First, an initial limited search of EMBASE and EBSCO across the databases has been undertaken to identify articles on this topic, followed by an analysis of the text words contained in the titles and abstracts, and of the index terms used to describe these articles. This will inform the development of a search strategy including identified keywords and index terms, which will be tailored for each database. The bibliographic databases will be systematically searched using block search with the Boolean operators AND, OR, NOT. A preliminary search strategy for EMBASE is detailed in Additional file 2.

We will search the databases: EMBASE, PubMed, CINAHL, Academic Search Premier, SCOPUS and Web of Science.

Unpublished studies from 1985 to the present date will be searched in OpenGrey and Google Scholar.

Text, opinion papers, websites, guidelines, policy documents and letters to editors will be searched across the websites of relevant institutions and organizations with an interest in patient safety, diagnosis and interprofessional teamwork.

Ongoing studies will be searched in the databases: NHS Research Register, Clinicaltrials.gov and OSF Registries for the last 5 years.

To ensure literature saturation, reference lists of included articles will be manually screened to identify additional studies. A systematic citation search will be performed to collect all articles that cite the original papers.

Studies published from 1985 to the present date will be included as the preliminary search revealed that the publication rate of literature concerning patient safety increased dramatically at this point.

All languages are accepted in the searches. Studies published in English, Norwegian, Swedish, and Danish will be included directly and studies in other languages will be translated, if considered relevant for inclusion in the scoping review.

The bibliographic databases will be searched again toward the end of the review process to ensure that the most recent studies are included.

### Study/source of evidence selection

Citations, abstracts, and full-text articles retrieved through the literature search will be uploaded to Covidence, an Internet-based bibliographic management software program operated by Veritas Health Innovation Ltd., Melbourne, Australia. Removal of duplicates and the screening process will be managed in Covidence based on the inclusion and exclusion criteria. To optimize and qualify the screening process, a calibration exercise, in which 50 titles and abstracts are read, discussed and assessed in agreement by the two authors (NLH & LNJ) responsible for screening, will be undertaken prior to the screening.

The selection process will be performed in two steps: first, screening by titles and abstracts, and second, screening of full text of reports selected by the first step. Both steps will be performed according to the inclusion and exclusion criteria and done independently by two authors (NLH & LNJ). Reasons for exclusion will be recorded and reported in the scoping review. Any disagreements that arise between the reviewers at each stage of the selection process will be resolved through discussion, or with additional reviewer or reviewers. The results of the search and the study inclusion process will be reported in full in the final scoping review and presented in a PRISMA-ScR flow diagram [6, 13].

### Data extraction/data charting

Data from eligible studies or other evidence sources will be extracted using a data extraction form designed for this study. The data extraction form will be designed using Covidence, and will include information on key study characteristics, e.g., title, authors, reference/source/year of publication, country, aim of study, setting, study design, sources of funding/competing interest; and specific details on all methods and metrics used, e.g., population, concept, context; study methods of significance to the review question; and specific objectives. The data extraction form will continuously be discussed and revised in an iterative process between reviewers throughout the data charting. A draft extraction form is provided in this protocol (see Additional file 3).

Two reviewers (NLH & LNJ) will independently extract and chart the data, followed by cross-checking and discussion of the results and continuous revision of the data extraction form in an iterative process. Any disagreement between the two reviewers will be resolved through discussion between all four co-authors. If necessary and appropriate, the authors of eligible studies will be contacted to request missing or additional data.

### Data analysis and presentation

The extracted data will be analysed in a qualitative synthesis and presented in diagrammatic or tabular form in a manner that aligns to the objective and scope of this scoping review. The tables and charts will report distribution of studies by year or period of publication, countries of origin, area of practice, research methods, and results. In the mapping of evidence, we will clearly indicate whether the evidence origins from published or unpublished studies, to check the effect of unpublished studies on the results. A narrative summary will accompany the tabulated and/or charted results and will describe how the results relate to the review’s objective and questions.

### Potential deviations from this protocol

Deviations from this protocol will be clearly mentioned in the review manuscript.

## Discussion

This scoping review aims to shed a most needed light on a possible solution to a present and severe challenge of diagnostic errors compromising patient safety. It will provide a solid foundation for further research and development in this significant field. In a long-term perspective, this scoping review will contribute to prevent diagnostic errors and improve patient safety, for adults and children with physical health issues.

To provide a unique and comprehensive mapping of the present published and unpublished knowledge and ongoing work on interprofessional diagnostic management teams and the derived effects on diagnostic safety, the scoping review will be performed in accordance with the recommended guidelines from JBI and PRISMA.

To achieve a high standard and quality, the systematic search strategy and search protocol for published peer-reviewed evidence will be developed in consultation with an experienced and skilled library and information specialist. Further, unpublished and ongoing studies will be retrieved thoroughly in relevant registries, databases and webpages. Literature saturation will be ensured by searching through the reference lists of all included material.

To our knowledge, this will be the first scoping review regarding interprofessional diagnostic teams and the derived effects on diagnostic safety and can therefore be a very important contribution to improve patient safety significantly during the diagnostic process.

### Supplementary Information

**Additional file 1.** Populated checklist for scoping review protocol. 

**Additional file 2.** Search terms for EMBASE. 

**Additional file 3.** Data extraction instrument – draft. 

## Data Availability

The datasets used and/or analysed during the current study are available from the corresponding author on reasonable request.

## Abbreviations

Dbio: Danish Association of Biomedical Laboratory Scientists [Danske Bioanalytikere]
IDMT: Interprofessional diagnostic management teams
JBI: Joanna Briggs Institute
LNJ: Lene Noehr-Jensen
NLH: Nicoline Lykke Hansen
NHS: National Health Service
OSF: Open Science Framework
PRISMA –ScR: Preferred Reporting Items for Systematic Reviews and Meta-Analysis for Scoping Review

## References

[1] National Academies of Sciences E. Improving diagnosis in health care. 2015. Cited 2021 May 4. Available from: https://www.nap.edu/catalog/21794/improving-diagnosis-in-health-care.

[2] Dansk Selskab for Patientsikkerhed, Patienterstatningen. Improving diagnosis in Danish health care [Veje til bedre diagnoser]. 2019. Cited 2021 May 4. Available from: https://patientsikkerhed.dk/content/uploads/2019/12/vejetilbedrediagnoserdec2019.pdf.

[3] Dansk Selskab for Patientsikkerhed. Improving Diagnosis in Danish Healthcare. Cited 2022 Dec 1. Available from: https://patientsikkerhed.dk/wp/wp-content/uploads/2023/04/improving-diagnosis-in-danish-health-care.pdf.

[4] Graber ML, Rusz D, Jones ML, Farm-Franks D, Jones B, Gluck JC (2017). The new diagnostic team. Diagnosis (Berl).

[5] Peters MDJ, Godfrey C, McInerney P, Khalil H, Larsen P, Marnie C (2022). Best practice guidance and reporting items for the development of scoping review protocols. JBI Evid Synth.

[6] Peters MDJ, Godfrey C, McInerney P, Munn Z, Tricco AC, Khalil H. Chapter 11: Scoping Reviews (2020 version). In: Aromataris E, Munn Z, editors. JBI Manual for Evidence Synthesis. JBI; 2020. Available from: https://synthesismanual.jbi.global. 10.46658/JBIMES-20-12.

[7] Munn Z, Peters M, Stern C, Tufanaru C, Mcarthur A, Aromataris E (2018). Systematic review or scoping review? Guidance for authors when choosing between a systematic or scoping review approach. BMC Med Res Methodol.

[8] Taberna M, Gil Moncayo F, Jané-Salas E, Antonio M, Arribas L, Vilajosana E, et al. The Multidisciplinary Team (MDT) Approach and Quality of Care. Frontiers in Oncology. 2020;10. Cited 2023 Oct 19. Available from: https://www.frontiersin.org/articles/10.3389/fonc.2020.00085. 10.3389/fonc.2020.00085 PMC7100151 32266126

[9] Verna R, Velazquez AB, Laposata M (2019). Reducing diagnostic errors worldwide through diagnostic management teams. Ann Lab Med.

[10] Wade J, Dean CL, Krummey SM, Roback JD, Sullivan HC (2020). How do I … implement diagnostic management teams in transfusion medicine?. Transfusion.

[11] Lubin IM, Astles JR, Shahangian S, Madison B, Parry R, Schmidt RL (2021). Bringing the clinical laboratory into the strategy to advance diagnostic excellence. Diagnosis (Berl)..

[12] World Health Organisation. ICD-11 for Mortality and Morbidity Statistics (Version : 01/2023). Available from: https://icd.who.int/browse11/l-m/en#/http%3a%2f%2fid.who.int%2ficd%2fentity%2f334423054.

[13] Tricco AC, Lillie E, Zarin W, O’Brien KK, Colquhoun H, Levac D (2018). PRISMA Extension for Scoping Reviews (PRISMA-ScR): Checklist and Explanation. Ann Intern Med.

