# Supplementary material for: Interprofessional diagnostic management teams: a scoping review protocol
Source: Syst Rev. 2023 Nov 22;12:223. doi: 10.1186/s13643-023-02391-2 (PMC10664282; doi:10.1186/s13643-023-02391-2)
Supplement: Supplementary file 2 — Additional file 2. Search terms for EMBASE. [file 13643_2023_2391_MOESM2_ESM.docx]

# Additional file 2: Search terms for EMBASE

Search terms for EMBASE, including limits:

(((diagnostic NEAR/7 process):ab,ti) OR (diagnostic NEAR/7 excellence) OR ((improv* NEAR/2 diagnos*):ab,ti) OR 'patient safety':ab,ti OR (('diagnostic error'/exp OR 'diagnostic error*':ab,ti) AND blind AND spot*) OR misdiagnosis:ab,ti OR 'missed opportunities in diagnosis' OR ((patient NEAR/4 hazard*):ab,ti)) AND diagnostic*:ab,ti AND (multidisciplinary:ab,ti OR interprofessional:ab,ti OR interdisciplinary:ab,ti OR (health:ab,ti AND professionals:ab,ti)) AND (team*:ab,ti OR collaborat*:ab,ti OR approach*:ab,ti OR practice*:ab,ti OR staff*:ab,ti) AND ([danish]/lim OR [english]/lim OR [norwegian]/lim OR [swedish]/lim) AND [1985-2021]/py
