# Supplementary material for: Interprofessional diagnostic management teams: a scoping review protocol
Source: Syst Rev. 2023 Nov 22;12:223. doi: 10.1186/s13643-023-02391-2 (PMC10664282; doi:10.1186/s13643-023-02391-2)
Supplement: Supplementary file 3 — Additional file 3. Data extraction instrument – draft. [file 13643_2023_2391_MOESM3_ESM.docx]

# Additional file 3: Data extraction instrument - draft

| Study Details | | | | | Inclusion/Exclusion Criteria | | | | | | | | | Details/Results extracted from source of evidence | | | | |
| --- | --- | --- | --- | --- | --- | --- | --- | --- | --- | --- | --- | --- | --- | --- | --- | --- | --- | --- |
| Title | Authors | Year | Country | Aim | Participants | | | | Concept DMT^1^ members | | Context | Type of evidence source | | Types and numbers of diagnostic errors before and after implementing diagnostic management teams | Impact on the patients and/or relatives^2^ | Impact on the healthcare professionals^3^ | Perception of participating in the diagnostic process by patients, relatives and healthcare professionals | Position on and perception of patient safety before and after implementation |
|  |  |  |  |  | # | Sex | Age | Somatic condition | # | Role | Setting | Study design | Type of evidence |  |  |  |  |  |
|  |  |  |  |  |  |  |  |  |  |  |  |  |  |  |  |  |  |  |
|  |  |  |  |  |  |  |  |  |  |  |  |  |  |  |  |  |  |  |
|  |  |  |  |  |  |  |  |  |  |  |  |  |  |  |  |  |  |  |
|  |  |  |  |  |  |  |  |  |  |  |  |  |  |  |  |  |  |  |
|  |  |  |  |  |  |  |  |  |  |  |  |  |  |  |  |  |  |  |
|  |  |  |  |  |  |  |  |  |  |  |  |  |  |  |  |  |  |  |
|  |  |  |  |  |  |  |  |  |  |  |  |  |  |  |  |  |  |  |
|  |  |  |  |  |  |  |  |  |  |  |  |  |  |  |  |  |  |  |

^1^diagnostic management team

^2^ e.g. level of patient satisfaction, trust in healthcare, self-efficacy

^3^ e.g. workflow, working environment, collaboration, job satisfaction
